# Predicting the Potential Geographic Distributions of Two Large Predatory Insects, *Microstylum dux* and *M. oberthurii* (Diptera: Asilidae), Under Climate Change: A Comprehensive Analysis Based on Optimised Biomod2 Ensemble Model

**DOI:** 10.3390/insects17050533

**Published:** 2026-05-21

**Authors:** Zhuoman Zhang, Zhendong Gao, Hu Li

**Affiliations:** Shaanxi Key Laboratory of Bio–Resources, State Key Laboratory of Biological Resources and Ecological Environment of Qinling–Bashan, Qinling–Bashan Mountains Bioresources Comprehensive Development C.I.C., School of Biological Science & Engineering, Shaanxi University of Technology, Hanzhong 723000, China; 19591167386@163.com (Z.Z.); soar0710@foxmail.com (Z.G.)

**Keywords:** *Microstylum dux*, *Microstylum oberthurii*, ensemble model, climate change, potential geographic distribution, niche dynamics

## Abstract

Climate change profoundly impacts insect distribution and ecological functions. For the predatory robber flies *Microstylum dux* and *M. oberthurii* (Diptera: Asilidae), clarifying their distribution and climatic responses is vital for natural enemy conservation and biological control. Using a parameter-optimized biomod2 ensemble model, we predicted their potential distributions under current and future climates, and analyzed key variables, centroid shifts, and niche dynamics. Current suitable habitats concentrate in southeast China and are scarce in the northwest. Future total suitable area remains stable but structurally reorganizes, with highly suitable habitats expanding and moderately suitable ones contracting. Key drivers are precipitation of the coldest quarter (bio19) and mean diurnal range (bio2). Habitat centroids migrate westward or southwestward with fluctuating range expansion. *M. dux* is a niche specialist (niche width = 0.257), while *M. oberthurii* is a generalist (niche width = 0.539). Their niche overlap shows non-linear “divergence-convergence-divergence” dynamics. This study supports natural enemy conservation and biological control strategy formulation.

## Simple Summary

Climate change is affecting where insects can live, including beneficial predatory insects that help control crop pests. This study investigated how two large predatory robber fly species in China might respond to future climate shifts. We wanted to predict where these insects could potentially live today and under different future climate scenarios, to see if their ranges would move, shrink, or expand. Using computer models and known insect locations, we found that areas highly suitable for these flies are currently in southeastern China. In the future, while the total area they could inhabit remains stable, the best-quality habitats are expected to expand, while moderately suitable areas will shrink significantly. Their overall living areas are also predicted to shift westward or southwestward. The two species have different environmental preferences, but the overlap in their preferred living conditions will change dynamically over time. Our findings provide crucial information for conserving these beneficial insects and planning how to use them for natural pest control in agriculture as the climate changes, which is important for protecting biodiversity and supporting sustainable farming.

## 1. Introduction

*Microstylum dux* Wiedemann (Diptera: Asilidae) and *Microstylum oberthurii* Wulp (Diptera: Asilidae) are large predatory natural enemy insects widely distributed in eastern and southern China. *M. dux* inhabits open woodland areas, with a body length that can reach up to 40 mm; *M. oberthurii* predominantly inhabits low to mid-elevation mountainous areas, with a body length of up to 45 mm, making it the largest known species of Asilidae in China [1,2]. Adult robber flies are agile aerial predators known for their rapid flight and aggressive hunting behavior, with a broad prey spectrum that includes various agricultural and forestry pests, such as leafhoppers, aphids, and planthoppers, making them potential agents for biological control [2]. However, adults also prey on beneficial insects like honeybees, which may cause disturbances in apiaries [3,4]. Existing studies have clearly confirmed that adult robber flies actively prey on pollinators such as honeybees and are an important group of natural enemies threatening colony health [5,6,7]. The larvae inhabit soil or decaying wood and are also predatory, feeding on the larvae or eggs of other insects [8,9]. They are recognized as important natural enemies of soil-dwelling pests such as white grubs (the larvae of scarab beetles) [10,11,12]. Consequently, they are considered to possess significant ecological value in controlling agricultural and forestry pests and in maintaining the balance of ecosystem food webs [2,4,13].

As a core component of many ecosystems, insects are highly sensitive to climate change, which has led to them being recognized as one of the animal groups most significantly affected [14,15,16]. Climate change, as a global environmental stressor, directly impacts insect physiology, growth, development, survival, reproduction, and geographical distribution through multiple pathways including rising temperatures, increased carbon dioxide concentrations, intensified droughts, and more frequent storms [17,18]. This disrupts their ecological functions, leading to reduced biodiversity, and habitat fragmentation [19]. Therefore, clarifying the responses of *M. dux* and *M. oberthurii* to climate change, along with their potential geographic distributions under current and future climate scenarios, is considered essential not only for assessing the sustainability of their roles as biocontrol resources, but also for providing a scientific basis to formulate forward-looking conservation strategies for natural enemy insects and to develop regional integrated pest management plans, thereby addressing agricultural economic risks posed by climate change.

Species Distribution Models (SDMs) serve as effective tools for projecting species distributions under climate change, finding widespread application across diverse fields including biogeography, ecology, and conservation biology [20,21]. By integrating species distribution data with high-resolution environmental layers, SDMs enable the effective prediction of potential geographical species ranges, which is crucial for deepening the understanding of species responses to climate change and for formulating scientific conservation and management strategies [22,23].

Currently, the most commonly used SDMs include over ten prevalent algorithms such as the Maximum Entropy Model (MAXENT), the Genetic Algorithm for Rule-set Prediction (GARP), Generalized Linear Models (GLM), and Random Forest (RF). However, the algorithmic limitations of single models (e.g., MAXENT or GLM) and their sensitivity to input data can potentially introduce bias into the results [24,25,26]. To enhance the robustness of predictions, the biomod2 ensemble modeling platform has been developed [27,28]. By integrating multiple algorithms, the biomod2 ensemble modeling approach can effectively overcome the algorithmic limitations and uncertainties associated with single models. This integration helps reduce prediction bias, overfitting, and noise, thereby significantly improving the accuracy, robustness, and reliability of the results [28,29]. The effectiveness of this framework has been extensively demonstrated through empirical applications in distribution prediction studies for insects, plants, and disease vectors [30,31,32].

As a major agricultural country, China faces substantial demands for pest control. However, existing research on *M. dux* and *M. oberthurii* has predominantly focused on morphological taxonomy and phylogenetic relationships, with few studies reported on the response of their geographical distributions to climate change [33,34,35]. Therefore, based on the geographical distribution records of *M. dux* and *M. oberthurii* in China and the latest climate data from the Coupled Model Intercomparison Project Phase 6 (CMIP6), this study employed the parameter–optimized biomod2 ensemble model to predict the potential geographical distributions of these two species under current climate conditions and three future climate scenarios (SSP1–2.6, SSP2–4.5, and SSP5–8.5). The aims of this study were: (1) to reveal the potential geographical distributions of *M. dux* and *M. oberthurii*; (2) to identify the key environmental drivers determining their distributions; (3) to predict changes in the extent of suitable habitats and the trajectory of distributional centroids under different future climate scenarios; and (4) to compare and analyze their niche widths and niche overlap dynamics to elucidate their ecological niche response characteristics. The findings are expected to fill the research gap concerning the ecogeographical distribution of these species and provide a theoretical foundation for the conservation, monitoring, and practical application of these natural enemy insects in biological control.

## 2. Materials and Methods

### 2.1. Species Distribution Data

The species occurrence data for *M. dux* and *M. oberthurii* used in this study were primarily obtained from the Global Biodiversity Information Facility (GBIF Occurrence Download. Available online: https://doi.org/10.15468/dl.ugtj5c (accessed on 26 November 2025); https://doi.org/10.15468/dl.smwms6 (accessed on 16 January 2026), specimen records collected by the research team, and related literature reports. To ensure data reliability, a strict filtering protocol was applied. Records lacking precise geographical information (e.g., missing longitude/latitude coordinates or specific location details) were manually removed. To avoid data duplication, and reduce spatial autocorrelation and sampling redundancy, the dplyr package (v1.1.4) [36] and spThin package (v0.2.0) [37] in R were used for data de-duplication and spatial sparsification processing. A 10 km spatial sparsification distance was adopted. This interval is a widely used threshold in species distribution modeling, which can eliminate spatial clustering phenomena while retaining sufficient samples for robust model calibration [37,38,39,40].

**Figure 1:**
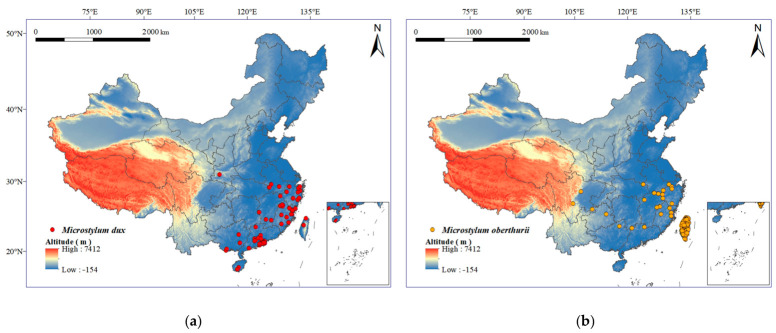
Geographical distribution records of (**a**) *M. dux* and (**b**) *M. oberthurii* within China. Note: This figure displays the locations of the 68 valid distribution points for *M. dux* (**a**) and the 99 valid distribution points for *M. oberthurii* (**b**) that were retained following the screening process. All geographical data within China referenced herein bear the approved map number: GS (2024) 0650.

For *M. dux*, 269 distribution records were obtained from GBIF Occurrence Download. Available online: https://doi.org/10.15468/dl.ugtj5c (accessed on 26 November 2025), while 7 valid distribution records were collected through field surveys and literature reviews, for a total of 276 occurrence records; for *M. oberthurii*, 476 distribution records were obtained from GBIF Occurrence Download. Available online: https://doi.org/10.15468/dl.smwms6 (accessed on 16 January 2026), while only 1 valid distribution record was collected through field surveys, for a total of 477 occurrence records. Following a filtering procedure, 68 and 99 valid occurrence points were retained for *M. dux* and *M. oberthurii*, respectively. The spatial visualization of the distribution points for both species was performed using ArcGIS (v10.8) (Figure 1).

### 2.2. Environmental Variables

The current (1970–2000) and future climate variable datasets were acquired from the WorldClim 2.1 database (https://www.worldclim.org/ accessed on 28 November 2025), at a spatial resolution of 2.5 arc-minutes. The datasets encompass 19 bioclimatic variables (bio1–bio19), which comprehensively represent key temperature and precipitation characteristics influencing species distributions [41]. Future climate projections were derived from the BCC-CSM2-MR (Beijing Climate Center climate system model version 2—medium resolution) climate model within the Coupled Model Intercomparison Project Phase 6 (CMIP6). This model is considered well-suited for regional simulations over China and has been demonstrated to reliably simulate long-term temperature and precipitation trends [42]. Three Shared Socioeconomic Pathways (SSPs) were selected: the sustainability pathway (SSP1–2.6), the middle road pathway (SSP2–4.5), and the fossil-fueled development pathway (SSP5–8.5), representing low, medium, and high greenhouse gas emission scenarios, respectively. These projections cover three future time periods: 2041–2060 (2050s), 2061–2080 (2070s), and 2081–2100 (2090s) [42,43]. All spatial data were processed and aligned using the WGS 1984 geographic coordinate system to provide a unified geospatial foundation for subsequent species distribution modeling.

In species distribution modeling, multicollinearity among environmental variables can lead to increased model complexity, and reduced predictive performance. Therefore, to eliminate the influence of multicollinearity on model stability, a stepwise screening strategy was employed. First, we used MAXENT model v3.4.4 to build an initial model in order to preliminarily assess the contribution of each climate variable [44,45,46]. Subsequently, Pearson correlation analysis and variance inflation factor (VIF) analysis were performed using the stats (v4.3.3) and usdm (v2.1–7) packages in R, and a heatmap of Pearson correlation coefficients (r) for climate factors was generated (Figure S1). We then used the “vifstep” function in the usdm package (v2.1–7) and set the threshold to VIF < 10 to further exclude variables exhibiting significant multicollinearity. The default threshold for the “vifstep” function in the usdm package is VIF > 10, which serves as a rule of thumb in statistical modeling for detecting severe multicollinearity; a VIF > 10 indicates a significant multicollinearity issue, whereas a VIF < 10 suggests no severe multicollinearity [47,48,49]. Subsequently, the pairwise Pearson correlation coefficients among the retained variables were verified, and the results confirmed that the maximum coefficient (|r|) was only 0.774, far below the threshold of |r| > 0.8 for determining high multicollinearity [47]. During the screening process, priority was given to retaining variables with higher contribution percentages (based on preliminary MAXENT modeling assessments) and clearer ecological relevance [50,51]. The final set of environmental variables used for biomod2 modeling is shown in Table 1. Multicollinearity among all retained variables was low, with a maximum VIF value of 4.81.

### 2.3. Model Construction and Evaluation

In this study, species distribution models (SDMs) were constructed using the ENMeval package (v1.1.5) and the biomod2 package (v4.2–6–2) within the R environment (v4.4.3) [52]. Initially, the ENMeval package was employed to optimize the regularization multiplier (RM) and feature combination (FC) parameters for the MAXENT and MAXNET (Maximum Entropy) models. The RM parameter was tested across a range from 0.5 to 4, incremented by 0.5. For the FC parameter, five feature classes were considered: Linear (L), Quadratic (Q), Hinge (H), Product (P), and Threshold (T). Six specific feature combinations (L, LQ, H, LQH, LQHP, LQHPT) were tested. The ENMeval package evaluated all 48 possible parameter combinations, then utilised the Akaike information criterion correction (AICc) to assess model complexity and fit. It will select the RM and FC values corresponding to delta.AICc = 0 for subsequent model operations [53].

After tuning the MAXENT and MAXNET model, we used the biomod2 package to select a total of 12 modeling algorithms: ANN (Artificial Neural Network), CTA (Classification Tree Analysis), FDA (Flexible Discriminant Analysis), GAM (Generalized Additive Model), GBM (Generalized Boosting Model), GLM, MARS (Multiple Adaptive Regression Splines), MAXENT, MAXNET, RF (Random Forest), SRE (Surface Range Envelop), and XGBOOST (eXtreme Gradient Boosting Training). We used the “bm_ModelingOptions” function in the biomod2 package to define model parameter options. For the MAXENT and MAXNET algorithms, which had been fine-tuned, we employed a custom “user.defined” parameter strategy. For the other algorithms, we adopted the widely used “bigboss” default parameter strategy to ensure computational stability and avoid over-tuning [28,53,54]. This hierarchical approach focuses tuning efforts on the models being optimized while maintaining robust, reproducible settings within the ensemble.

Pseudo-absence points were generated across the entire territory of China (4°–54° N, 73°–135° E, including the islands in the South China Sea), consistent with the overall boundaries of our study area. Pseudo-absence points were generated using a random strategy. This approach is suitable for the two robber fly species present, as their relatively broad geographic and habitat distributions reduce the risk of environmental undersampling when using random background point extraction. In line with common practices for small sample sizes and established methodological guidelines [55,56], and to accommodate the requirements of different algorithms within the biomod2 ensemble framework (e.g., regression algorithms needing numerous background points versus machine learning algorithms favoring a 1:1 ratio), a presence-to-pseudo-absence ratio higher than 1:1 was defined. Specifically, 500 pseudo-absence points (a ratio of approximately 1:7.4) were selected for *M. dux*, and 900 pseudo-absence points (a ratio of approximately 1:9.1) for *M. oberthurii*. This setting ensured adequate environmental background coverage for model calibration while minimizing predictive bias due to severe sample-size imbalance. For each species, 75% of the species occurrence records were randomly allocated to the training dataset, with the remaining 25% were reserved for model testing. Each model was run with 10 replicates, resulting in a total of 120 model runs per species, calculated as the product of the number of single models, the number of pseudo-absence sets, and the number of replicates.

Model accuracy and reliability were assessed using the True Skill Statistic (TSS) and the Area Under the Receiver Operating Characteristic Curve (AUC). The AUC value ranges from 0.5 to 1.0, where 0.5 indicates a performance no better than random and 1.0 indicates perfect predictive ability. An AUC value above 0.8 is generally considered to indicate good to excellent predictive performance, while a value below 0.7 suggests poor performance. The TSS, which represents the net prediction success rate on observed data, ranges from −1 to 1. A TSS value greater than 0.7 is considered to reflect high model accuracy, whereas a value below 0.5 indicates low accuracy [57]. For the final ensemble modeling, only models that met strict threshold criteria were retained: for *M. dux*, models required an AUC ≥ 0.90 and TSS ≥ 0.80; for *M. oberthurii*, the thresholds were set at AUC ≥ 0.95 and TSS ≥ 0.80. The final ensemble model was constructed using the weighted mean method (EMwmean).

### 2.4. Habitat Suitability Classification and Spatiotemporal Change Analysis

The continuous probability distribution maps were converted into binary maps (suitable vs. non-suitable areas) using the threshold that maximized the TSS. This TSS-maximized threshold was selected not only for its high predictive accuracy in species distribution modelling, but also for its ecological and biological rationality: it effectively delineates the habitat suitability boundary of robber flies by balancing the omission of true suitable habitats and the commission of unsuitable areas [58]. This binarization facilitated subsequent area quantification and spatial pattern analysis. The ensemble model predictions were then imported into ArcGIS 10.8, where the natural breaks (Jenks) classification method was applied to categorize habitat suitability into four distinct classes: unsuitable, slightly suitable, moderately suitable, and highly suitable areas [59]. The natural breaks method is capable of more accurately reflecting the spatial heterogeneity of suitability indices and revealing the genuine transition boundaries between different ecological zones [60,61], which thus provides a reliable basis for subsequent ecological risk assessment and biodiversity conservation. This approach not only enhances the objectivity and reliability of the classification, but also facilitates an in-depth analysis of the suitability conditions for various ecological regions [31]. Critically, all classification thresholds (both for binarization and for the four suitability classes) were derived from the current model predictions and held fixed when applied to future projections, ensuring direct comparability of habitat classes across time periods.

To visualize the directional trends in potential habitat shifts, the SDMtoolbox (v2.6) (http://www.sdmtoolbox.org/ accessed on 24 November 2025) was utilized to calculate the centroid positions of suitable areas for each time period [62]. Centroid migration trajectories and standard deviational ellipses were generated. The impact of climate change on the distribution of *M. dux* and *M. oberthurii* was quantified by measuring the migration distance and direction of these centroids along the longitude and latitude axes. Additionally, parameters of the standard deviational ellipses, including area, ellipticity, and the rotation angle of the major axis, were calculated. Changes in the spatial extent of habitats were assessed by computing the absolute and percentage changes in the area of each suitability class across different time periods and climate scenarios. All spatial analyses, including area calculations, change assessments, centroid trajectory mapping, and standard deviational ellipse visualizations, were performed within the ArcGIS 10.8 environment.

### 2.5. Analysis of Multivariate Environmental Similarity Surface (MESS)

Multivariate Environmental Similarity Surfaces (MESS) analysis was conducted using the predicts package (v0.1–19), which is the successor package to the original dismo package and features a more concise syntax and does not rely on a Java environment [63]. MESS analysis provides a quantitative basis for assessing the reliability of species distribution models when they are extrapolated from current to future climatic conditions. Its core function is to identify areas where model predictions may become unreliable when applied to novel environments (extrapolation). This is achieved by comparing the environmental variables in the target region with those in the training region, generating a similarity map. Areas with negative values on this map represent “high-risk” extrapolation zones where model results should be interpreted with caution [63,64,65,66].

### 2.6. Niche Dynamics Analysis

To systematically evaluate the niche dynamics of *M. dux* and *M. oberthurii* across different time periods and climate scenarios, a principal component analysis (PCA) of the environmental space was conducted. This analysis focused on quantifying niche breadth, niche volume (representing the 95% core area), and niche overlap between the species. The method proposed by Broennimann et al. [67], which is based on a PCA framework, was adopted. Within this two-dimensional PCA environmental space, niche breadth (*B*) was defined as the mean squared Euclidean distance of each occurrence point from the origin. Niche breadth indices, including the Shannon niche index and Levins niche index, were considered. The analysis was performed using the ecospat package (v4.1.3) in R, which implemented the Levins method for calculating niche breadth [68]. The Levins niche breadth index was computed using the following formula:

(1) $$B_{i}=\frac{1}{\sum_{j=1}^{r}(P_{ij}{)}^{2}}$$

In Equation (1), *Bi* represents the niche breadth of species *i*, *Pij* denotes the proportion of occurrences of species *i* in a given locality or environmental grid cell *j*, and *r* is the total number of environmental grid units. 

To evaluate the potential spatial overlap, competition potential, and the impact of climate change on distributional shifts and interspecific relationships between *M. dux* and *M. oberthurii*, niche overlap was quantified under different periods and climate scenarios. This analysis was performed using the MASS package (v7.3–65) in R. Two widely used similarity metrics, Schoener’s *D* and the Hellinger-based *I* index, were calculated to quantify the degree of niche overlap between the two species. These indices are defined by the following formulas:

(2) $$D\;=\;1-\frac{1}{2}\sum_{j=1}^{r}|P_{1j}-P_{2j}|$$

(3) $$I=\frac{\sum_{j=1}^{r}P_{1j}P_{2j}}{\sqrt{(\sum_{j=1}^{r}P_{1j}{)}^{2}(\sum_{j=1}^{r}P_{2j}{)}^{2}}}$$

In Equations (2) and (3), *P*1*j* and *P*2*j* represent the Hellinger-transformed proportional utilization values of species 1 and species 2 in environmental grid unit *j*, and *r* is the total number of environmental grid units.

The values of *D* and *I* range from 0 to 1. The closer the value is to 1, the greater the degree of niche overlap and the stronger the stability, indicating that species may face similar environmental conditions and compete for similar resources. Conversely, the closer the value is to 0, the lower the degree of overlap, suggesting differences in niche preferences or significant niche displacement [69].

## 3. Results

### 3.1. Model Accuracy Assessment

**Figure 2:**
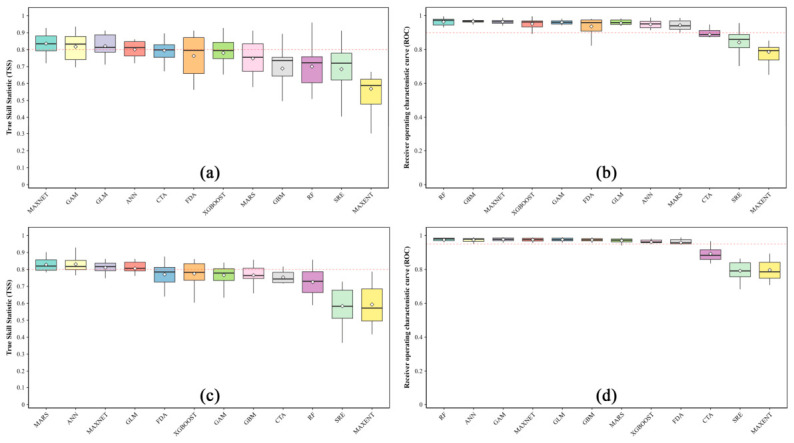
Performance evaluation of 12 single models for (**a**,**b**) *M. dux* and (**c**,**d**) *M. oberthurii* based on TSS and AUC. Note: Boxplots show model performance (TSS in (**a**,**c**); AUC in (**b**,**d**)) across 10 replicate runs. Red dashed lines indicate inclusion thresholds for the ensemble: AUC ≥ 0.90 and TSS ≥ 0.80 for *M. dux*; AUC ≥ 0.95 and TSS ≥ 0.80 for *M. oberthurii*.

During the construction of individual models, parameter tuning for key models was performed using the ENMeval package. For *M. dux*, the MAXENT and MAXNET models were optimized, with the final feature combination set to LQH and the regularization multiplier set to 2.0 (Figure S2). For *M. oberthurii*, the MAXNET model was optimized, configuring it with the LQH feature combination and a regularization multiplier of 1.0 (Figure S3). Subsequently, based on the TSS and AUC scores of the individual models, the best-performing models were selected for ensemble modeling. For *M. dux*, four algorithms: MAXNET, GLM, GAM, and ANN were integrated. For *M. oberthurii*, the ensemble was constructed using the ANN, MARS, MAXNET, and GLM. (Figure 2, Table S1). The results showed that the ensemble model for *M. dux* achieved a TSS score of 0.889 and an AUC score of 0.981 (Table S1), both superior to any single model. Similarly, the ensemble model for *M. oberthurii* attained a TSS score of 0.887 and an AUC score of 0.988 (Table S1), also outperforming all single models.

### 3.2. Potential Geographic Distribution Patterns and Spatiotemporal Dynamics of M. dux and M. oberthurii

**Figure 3:**
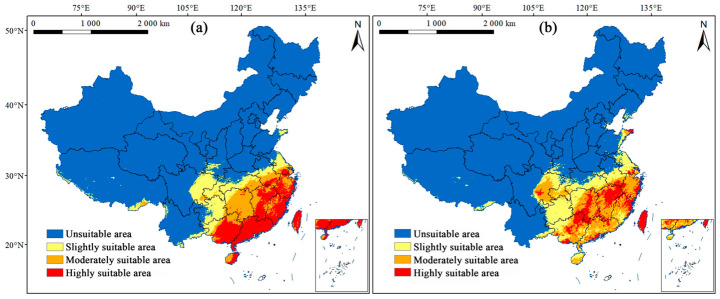
Current potential geographic distribution of (**a**) *M. dux* and (**b**) *M. oberthurii* in China. Note: Habitat suitability is classified into four grades based on the Jenks natural breaks method: blue = unsuitable area, yellow = slightly suitable area, orange = moderately suitable area, and red = highly suitable area.

**Figure 4:**
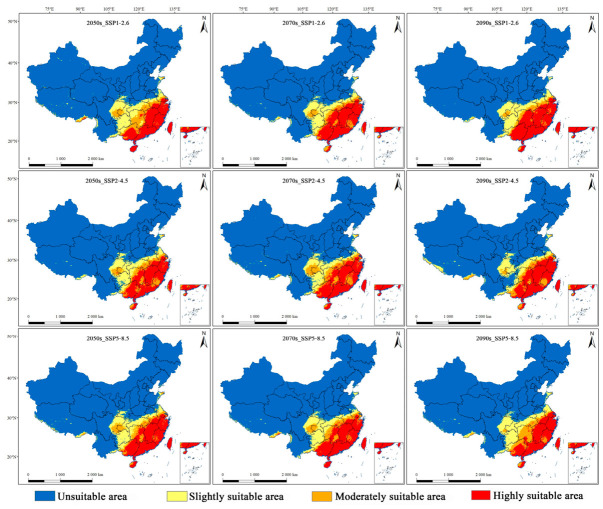
Predicted potential geographic distributions of habitat suitability for *M. dux* under different periods and climate scenarios. Note: The maps show suitability grades (unsuitable: blue; slightly suitable: yellow; moderately suitable: orange; highly suitable: red) across three time periods (2050s, 2070s, 2090s) and three SSP scenarios (SSP1–2.6, SSP2–4.5, SSP5–8.5).

**Figure 5:**
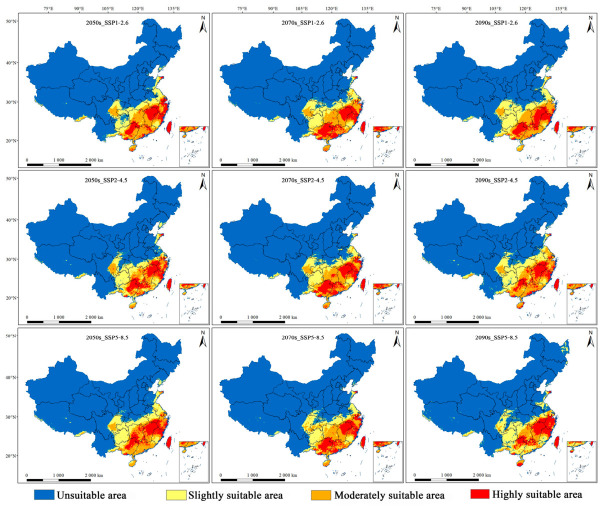
Predicted potential geographic distributions of habitat suitability for *M. oberthurii* under different periods and climate scenarios. Note: The maps show suitability grades (unsuitable: blue; slightly suitable: yellow; moderately suitable: orange; highly suitable: red) across three time periods (2050s, 2070s, 2090s) and three SSP scenarios (SSP1–2.6, SSP2–4.5, SSP5–8.5).

**Figure 6:**
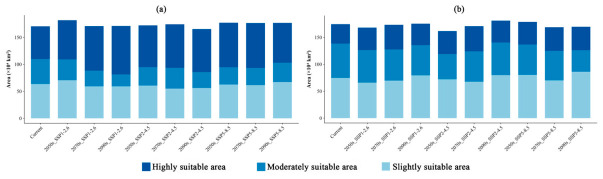
Areas of potential habitat for (**a**) *M. dux* and (**b**) *M. oberthurii* across different suitability classes under current and future climate scenarios. Note: The stacked bar charts display the total suitable habitat area (×10^4^ km^2^) partitioned into three classes: highly suitable area (dark blue), moderately suitable area (medium blue), and slightly suitable area (light blue). The *x*-axis includes the current climate and three future periods (2050s, 2070s, 2090s) under three SSP scenarios (SSP1–2.6, SSP2–4.5, SSP5–8.5).

**Figure 7:**
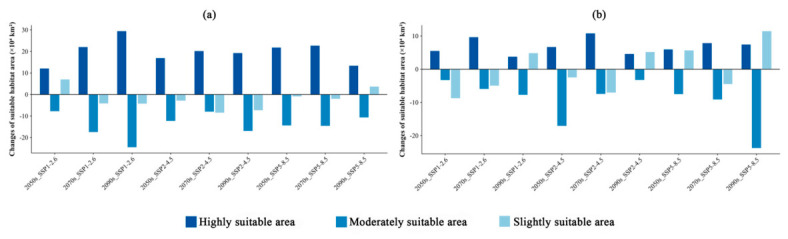
Changes in the area of potential suitable habitat for (**a**) *M. dux* and (**b**) *M. oberthurii* across different suitability levels under current and future climate scenarios. Note: Bar charts show the change in suitable habitat area (×10^4^ km^2^) relative to the current climate baseline. Positive values indicate an increase, while negative values indicate a decrease. Habitat suitability classes are represented by three shades of blue: dark blue = highly suitable area, medium blue = moderately suitable area, and light blue = slightly suitable area. The *x*-axis shows future climate scenarios (SSP1–2.6, SSP2–4.5, SSP5–8.5) across three time periods (2050s, 2070s, 2090s).

The potential geographical distributions of *M. dux* and *M. oberthurii* in China under current and future climate scenarios were visualized (Figure 3, Figure 4 and Figure 5). In the figures, red, orange, yellow, and blue areas represent highly, moderately, lowly, and non-suitable habitats, respectively. Statistical changes in the area of suitable habitats across different periods and scenarios are shown in Figure 6 and Figure 7, and Table S2.

Under current climate conditions, the potential suitable habitats for both species exhibited a similar pattern characterized by “concentration in the southeast and absence in the northwest.” The highly and moderately suitable habitats were primarily concentrated in central-eastern, southern, and southeastern coastal China (Figure 3). For *M. dux*, the total suitable area was 170.49 × 10^4^ km^2^. The highly suitable habitat (60.55 × 10^4^ km^2^) formed a contiguous block distributed across parts of South China, East China, and the entire island of Taiwan. The moderately suitable habitat (46.42 × 10^4^ km^2^) encircled the periphery of the highly suitable area, extending northwestward to cover most of Hunan, eastern Hubei, and southern Anhui. The slightly suitable habitat (63.52 × 10^4^ km^2^) further extended its coverage to areas including central-eastern Guizhou, central-southern Chongqing, and eastern Sichuan. The total suitable area for *M. oberthurii* was approximately 174.64 × 10^4^ km^2^. Its highly suitable habitat (36.09 × 10^4^ km^2^) appeared as scattered patches. The moderately suitable habitat (63.87 × 10^4^ km^2^) was more extensive, spanning multiple provinces across South China, East China, and Central China. The slightly suitable habitat (74.68 × 10^4^ km^2^) extended northwestward, reaching areas such as Guizhou, eastern Sichuan, and Jiangsu. The warm and humid climate in these regions provides suitable habitat conditions for both species.

Under different future climate scenarios, the total suitable area for both species remained relatively stable, while the internal composition of their suitable habitats (i.e., the relative proportions and spatial arrangement of suitability classes) underwent significant reorganization. The areas of highly suitable habitats generally expanded, whereas the moderately suitable habitat experienced the most severe contraction (Figure 6 and Figure 7, and Table S2). Specifically, for *M. dux*, the highly suitable area increased significantly across all scenarios (by 19.96% to 48.58%), with the most notable expansion projected for the end of the century (2090s) under the SSP1–2.6. In contrast, the moderately suitable area was substantially reduced (by 16.70% to 52.73%), with its area projected to be more than halved under the SSP1-2.6 (Figure 7a, Table S2a). *M. oberthurii* exhibited a similar trend: its highly suitable area continued to increase (by 10.38% to 29.88%), and its slightly suitable area also expanded (with an increase of up to 15.33% under the SSP5–8.5 scenario). Its moderately suitable area was also severely reduced (by 5.03% to 37.14%), with an estimated one-third of this habitat type projected to disappear by the 2090s under the SSP5–8.5 (Figure 7b, Table S2b).

In summary, under future climate change, the potential distributions of both *M. dux* and *M. oberthurii* exhibit a common pattern: an increase in highly suitable area, a severe decrease in moderately suitable area, a tendency for slightly suitable area to expand, and only minor fluctuations in the total area.

### 3.3. Climatic Variable Importance Analysis

To assess the influence of the input climatic variables on the distribution predictions for *M. dux* and *M. oberthurii*, and to gain deeper insights into the relationship between climatic variables and habitat suitability, the relative importance of the input climatic variables was quantitatively evaluated using the ensemble model outputs from biomod2 and visualized. Additionally, response curves for the top two most influential variables were plotted for each species (Figures S4 and S5). For *M. dux*, bio19 was identified as the most important limiting factor; bio2 was a secondary influential factor (Figure S4a). For *M. oberthurii*, the most important factor was bio2, while bio19 served as a secondary factor (Figure S4b).

The response curves of *M. dux* and *M. oberthurii* to key climatic factors show that both prefer environments with humid cold seasons and smaller diurnal temperature ranges, albeit with specific differences in their responses. The habitat suitability for *M. dux* showed a distinct saturating increase in relation to bio19. Suitability was low when precipitation was below 200 mm, rose rapidly once this threshold was exceeded, and stabilized at a high level after reaching 300–350 mm. Concurrently, its suitability peaked when bio2 was between 5–7 °C and declined significantly when the temperature range exceeded 8 °C (Figure S5a,b). *M. oberthurii* exhibits similar response trends to the same factors but with lower dependency, consistently showing lower habitat suitability than *M. dux* under identical precipitation conditions. Additionally, its optimal diurnal temperature range is slightly broader than that of *M. dux* (approximately 6–8 °C) (Figure S5c,d).

### 3.4. Centroid Shifts and Evolution of Suitable Habitat Distribution

The distance and direction of centroid migration reveal the shifting trends of the overall distribution center of habitats, while changes in ellipse parameters reflect the dynamic alterations in the extent and orientation of suitable habitats.

**Figure 8:**
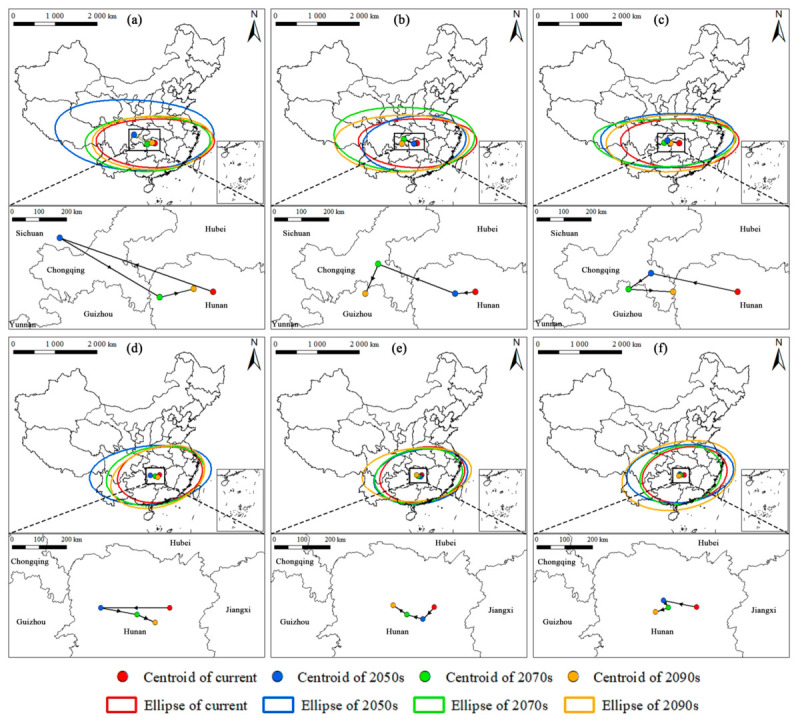
Future suitability ellipse variations and centroid migration trajectories of (**a**–**c**) *M. dux* and (**d**–**f**) *M. oberthurii* under different SSP scenarios and time periods. (**a**,**d**) SSP1–2.6; (**b**,**e**) SSP2–4.5; (**c**,**f**) SSP5-8.5. Note: The colored ellipses represent the 95% spatial range of suitable habitats, while the points and connecting lines indicate the centroid migration trajectories. Red = current period, blue = 2050s, green = 2070s, and orange = 2090s. The insets provide a zoomed–in view of the centroid shifts within the core suitable region.

The distribution centroid of *M. dux* exhibited an overall westward shift with substantial displacement magnitudes (ranging from 65.99 to 529.22 km) and showed pronounced fluctuations across different scenarios, indicating that its habitats are undergoing long-distance, unstable, and leapfrog adjustments. The maximum displacement of 529.22 km towards the northwest occurred under the 2050s_SSP1–2.6. The area of the suitable habitat ellipse changed significantly, particularly under the SSP1–2.6, showing a pattern of initial substantial expansion followed by contraction. Concurrently, the distribution shape became more elongated in the future. Nevertheless, the overall east–west extended spatial pattern remained highly stable (Figure 8a–c, Table S3a).

In contrast, the distribution center of *M. oberthurii* displayed a southwestward migration with minor fluctuations (ranging from 29.64 to 223.50 km), reflecting greater spatial stability. Its most significant displacement was a 223.50 km westward shift under the 2050s_SSP1–2.6. The area of its suitable habitat ellipse generally showed an expansion trend, with a maximum increase of approximately 64.3% compared to the current area. The distribution shape also tended to become more elongated, with the highest eccentricity (0.7651) observed under the 2050s_SSP1–2.6 scenario. The dominant east-west spatial pattern, however, remained very stable (Figure 8d–f, Table S3b).

### 3.5. Multivariate Environment Similarity Surface (MESS) Analysis

**Figure 9:**
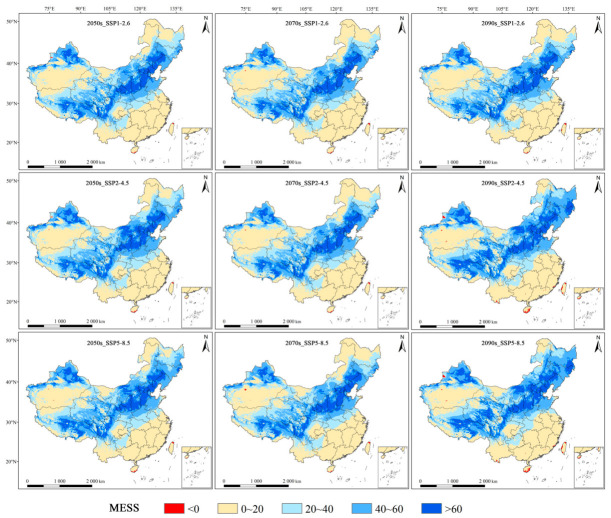
MESS analysis for *M. dux* under different future climate scenarios. Note: Red indicates a negative multivariate similarity (significant environmental differences, high extrapolation risk); other colors indicate a positive multivariate similarity (similar environments, low extrapolation risk).

**Figure 10:**
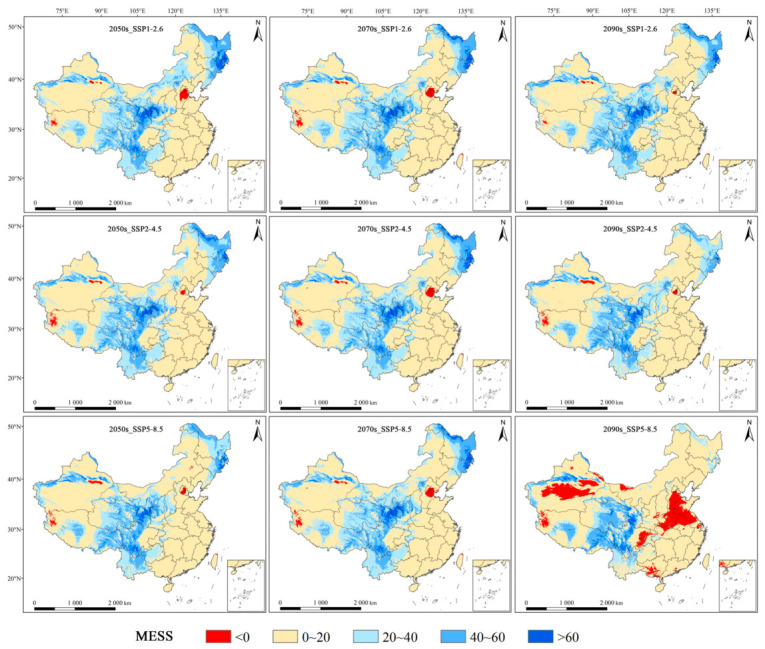
MESS analysis for *M. oberthurii* under different future climate scenarios. Note: Red indicates a negative multivariate similarity (significant environmental differences, high extrapolation risk); other colors indicate a positive multivariate similarity (similar environments, low extrapolation risk).

For *M. dux*, the mean multivariate similarity values across all future periods and climate scenarios were relatively high, ranging from 29.5 to 34.7, with minor variations observed between different scenarios. The highest mean value (34.7) was recorded for the 2090s under SSP2–4.5, while slightly lower values (29.5–29.6) were found for the 2070s under SSP1–2.6 and SSP5–8.5. The proportion of areas with negative multivariate similarity (climate anomaly areas) fluctuated slightly between 0.02% and 0.09% (Table S4a). These very limited climate anomaly areas were predominantly scattered in a few small, isolated regions to the south and east of the current highly suitable habitat (Figure 9). For *M. oberthurii*, the mean multivariate similarity values under all future climate scenarios, ranging from 11.2 to 18.9, were significantly lower than those for *M. dux*. The highest similarity (and lowest climate anomaly) was observed for the 2050s under SSP1–2.6, whereas the lowest similarity (and highest climate anomaly) was recorded for the 2090s under SSP5–8.5. The proportion of areas with negative multivariate similarity varied between 0.09% and 3.24% (Table S4b). Most climate anomaly areas were located at the periphery of the current suitable habitat, with a smaller portion extending into the southwestern and northwestern sectors of the current range (Figure 10).

### 3.6. Assessment of Ecological Niche Overlap and Dynamic Analysis of M. dux and M. oberthurii

Under current climatic conditions, the niche width of *M. dux* was 0.257, while that of *M. oberthurii* was 0.539, suggesting that the former is a niche specialist and the latter a generalist, reflecting a fundamental difference in their breadth of environmental resource utilization (Figure S6, Table S5). Projections under future climate scenarios revealed divergent trends: the niche width of *M. dux* was predicted to broaden across all scenarios, particularly increasing to 0.318 by the 2050s under the SSP5–8.5. In contrast, the niche width of *M. oberthurii* fluctuated around its current value, reaching approximately 0.57 by the end of the century under both the SSP2–4.5 and SSP5–8.5 (Table S5). Under the current climate conditions, the ecological niche overlap values for the two species are *D* = 0.464 and *I* = 0.764. Under different future scenarios, the overlap exhibited fluctuations: it peaks during the 2070s under multiple scenarios (*D* = 0.518, *I* = 0.806); it reaches its lowest point in the 2050s under the high emissions scenario (SSP5–8.5) (*D* = 0.440, *I* = 0.749). By the end of the century (2090s), although the overlap slightly declines from its peak, it generally remained close to or above the current levels (Table S5).

PCA results revealed a distribution pattern of “overall separation with local overlap” for the two species under the current climate (Figure S7). Under future climate scenarios, a dynamic pattern of niche overlap was observed, characterized by “initial divergence (2050s), subsequent convergence (2070s), and renewed divergence (2090s)”. The greatest degree of separation occurred under the 2050s_SSP5–8.5 scenario (Figure S8). Key bioclimatic variables, such as bio19, bio15 (Precipitation seasonality), bio2, and bio14 (Precipitation of driest month), consistently demonstrated high loading values on PC1 and PC2 (Figure S9).

## 4. Discussion

### 4.1. Advantages and Reliability of the Modeling Approach

The evaluation results of the ensemble model employed in this study exhibited extremely high accuracy (AUC > 0.98, TSS > 0.88), which fully validated the reliability of the species distribution predictions based on the ensemble modeling approach. This also confirmed the advantage of integrating multiple algorithms to construct an ensemble model in reducing random errors and enhancing robustness and generalization ability [28].

### 4.2. Spatiotemporal Distribution Dynamics and Environmental Drivers of the Suitable Habitats for M. dux and M. oberthurii

Accurately predicting the potential geographical distribution of natural enemy insects forms the foundation for assessing their biocontrol potential and formulating conservation strategies. The results of this study indicate that under the current climate, the suitable habitats for both species are concentrated in southeastern China, which is closely associated with the warm and humid climate of the East Asian monsoon region, suggesting that hydrothermal conditions serve as key limiting factors [70,71]. Under future climate conditions, the total suitable area for both species shows little change. However, an expansion of highly suitable habitat coexists with a contraction of moderately suitable habitat, indicating that climate change primarily drives a structural reorganization of habitat suitability rather than a simple expansion or contraction of range. Moderately suitable habitats, which function as transitional zones, are highly sensitive to climatic fluctuations and are prone to transformation. This reflects the gradual differentiation of suitable habitats into two types: highly suitable and marginally suitable. Such structural changes may exacerbate habitat fragmentation, thereby affecting connectivity among populations and gene flow [72,73].

Moreover, the variability in the direction of the centroid migration trajectories of both species indicates a potential shift towards regions characterized by more favorable hydrothermal conditions and increased environmental stability. This phenomenon precisely reveals the complexity of species adaptation strategies. Concurrently, standard deviational ellipse analysis indicates that future habitats may exhibit a concentrated and elongated distribution pattern along topographic or climatic corridors. These corridors are typically characterized by moderate temperature fluctuations and favorable moisture conditions, which can mitigate the extreme impacts of regional climate change.

Notably, *M. dux* was found to be more sensitive to precipitation, showing a saturating response curve, whereas *M. oberthurii* exhibited a stronger response to temperature range, reflecting its higher requirement for temperature stability. These subtle differences in environmental preference may constitute one of the mechanisms enabling their coexistence within similar distributional ranges. It is noteworthy that under future high-emission scenarios (SSP5–8.5), the synergistic effect of reduced precipitation and increased temperature range may intensify the spatial heterogeneity of habitat suitability, promote the expansion of marginal habitats, and consequently exacerbate population vulnerability.

### 4.3. Assessment of Model Extrapolation Uncertainty and Prediction Reliability

The MESS analysis results reveal that the reliability of the model predictions for the future distributions of the two natural enemy insects is fundamentally different, which primarily stems from varying degrees of model extrapolation risk. For *M. dux*, the predicted future areas are characterized by a high and stable mean multivariate environmental similarity across different scenarios, coupled with an extremely low proportion of climate anomaly areas. This indicates that the climatic conditions in its predicted potential distribution areas are highly similar to those of the current distribution areas used for model training. Consequently, the model uncertainty is lower, and the prediction results are considered to have relatively high reliability. In contrast, for *M. oberthurii*, the mean multivariate environmental similarity is significantly lower, and the proportion of climate anomaly areas substantially exceeds that of *M. dux*, with a notable deterioration especially under the long-term high-emission scenario (SSP5–8.5–2090s). However, these climate anomaly areas are primarily located at the periphery of the current suitable habitat, with a smaller portion involving areas in the southwest and northwest of the current range (Figure 10). Nevertheless, predictions for both species should be treated with caution [65,74].

### 4.4. Niche Dynamics and Non-Linear Responses of Interspecific Relationships

Niche breadth analysis revealed that *M. dux* is a specialist species, whereas *M. oberthurii* is a generalist species, which is consistent with their respective geographical distribution patterns: the suitable habitat of the specialist species is more concentrated, while that of the generalist species is more widespread. Although the niche-specialized *M. dux* exhibits a degree of niche plasticity, its adaptive range is relatively narrow, resulting in higher survival risk. In contrast, the niche-generalized *M. oberthurii*, benefiting from its broader fundamental niche width, demonstrates stronger adaptive potential and greater distribution stability under various future climate scenarios.

The niche overlap metrics (*D* and *I*) exhibited fluctuations across different time periods and climate scenarios, indicating that the impact of climate change on interspecific relationships is not a simple monotonic linear process [75]. Under the medium emission scenario in the medium term (2070s), niche overlap reached its peak, which may be attributed to niche convergence driven by climatic forcing [76]. In contrast, a decrease in overlap was observed in the near term (2050s) under the high emission scenario, suggesting that intense short-term climate perturbations may promote niche differentiation [75]. The PCA biplot further confirmed the ecological niche pattern of “overall separation with local overlap” between the two species, reflecting their potential for coexistence alongside trade-offs in resource utilization. These dynamic variations support the theoretical interplay between niche conservatism and niche plasticity [77,78]. Temperature and precipitation gradients are the most stable dominant factors shaping the niche spatial patterns of both species.

Despite the significant difference in niche width between the specialist *M. dux* and the generalist *M. oberthurii*, a sustained and relatively high niche overlap is found between them under both current and future conditions. These findings reveal that the impact of climate change on species niches is not a simple linear projection but a complex, non-linear dynamic adaptation process. This emphasizes the importance of considering niche dynamic processes when predicting species distributions and formulating conservation strategies. This dynamic overlap characteristic, combined with their distribution patterns, provides crucial quantitative insights for understanding their interspecific relationships, community stability, and potential future coexistence trajectories.

### 4.5. Tentative Implications for Conservation and Biological Control Considerations

Based on the systematic projections of the distribution patterns for *M. dux* and *M. oberthurii* under current and future climatic conditions, this study elucidates their potential in biological control as natural enemies, as well as the associated ecological risks. Currently, the highly suitable habitats are primarily located in major agricultural regions such as South China and East China. Future projections indicate a potential westward or southwestward shift of suitable areas, which may provide novel natural enemy resources for local pest management. However, because our models do not account for prey availability, dispersal capacity, land use, vegetation, soil, population dynamics, or interspecific interactions, any inference about actual biological control efficacy is speculative. Rather than prescribing management actions, we recommend that future research prioritise: (1) field validation of predation pressure; (2) integration of non-climatic factors into distribution models; (3) assessment of dispersal limitations; and (4) long-term population monitoring. Until such data are available, our results should be used to generate hypotheses and guide further ecological investigation, not as direct guidelines for biological control deployment.

### 4.6. Study Limitations and Future Perspectives

Although this study has revealed the distribution patterns and niche differentiation of the two *Microstylum* species under current and future climate conditions through an ensemble modeling approach, several limitations remain. These limitations also highlight key directions for future research.

Firstly, the models relied primarily on climatic data and did not fully account for non-climatic factors such as vegetation type, soil properties, and human activities, nor did they consider future landscape changes (e.g., land use/land cover change, urbanization, deforestation). This may lead to an underestimation of the species’ adaptive potential at local scales and an overestimation of climate-driven range shifts when landscape barriers exist. Future studies should integrate multi-dimensional elements, including climate, topography, soil, dynamic land-use scenarios, and anthropogenic disturbance, to construct a more comprehensive ecological framework. Secondly, an asymmetric parameter optimization strategy was applied to different species distribution model algorithms in this study. Although this approach was based on a trade-off between practical computational efficiency and model sensitivity, it may still introduce potential bias into the final ensemble predictions. Future studies, under conditions of sufficient computational resources, could conduct uniform and systematic parameter tuning for all modeling algorithms involved, thereby further enhancing the consistency and robustness of ensemble forecasts.

Thirdly, the interpretation of future suitable habitats requires caution. The uncertainty in future distribution projections mainly derives from four aspects. First, future climate scenarios entail inherent uncertainties. Although multiple SSP pathways were adopted to simulate diverse developmental trends, they merely represent reasonable future speculations [74]. Second, model extrapolation inevitably brings predictive risks, and model performance declines sharply when predicting climatic environments far beyond current conditions [65,79], which has been verified by the MESS analysis in this study [63]. Third, all future projections were generated based on a single CMIP6 global climate model instead of multi-model ensembles, which limits the generalizability of our findings. We clarify that our outcomes only stand for scenario-specific predictions under the selected climate model, rather than robust multi-model forecasts. Fourth, our model validation based on random training-test partitioning may overestimate actual predictive capability. Given that species occurrence records are often spatially clustered due to sampling constraints, spatial autocorrelation between training and test datasets tends to inflate ensemble AUC and TSS values, thus overrating model reliability. Hence, relevant conservation and management practices based on our findings ought to fully acknowledge such limitations, and long-term field monitoring is suggested to verify predictions and optimize management strategies. Further studies can incorporate multiple climate models and adopt spatially stratified validation such as block cross-validation to acquire more objective, universal, and stable prediction results.

Fourthly, biological processes such as species dispersal capacity, population dynamics, and interspecific interactions were not considered in the models. This could result in an overly optimistic assessment of the actual accessibility of future suitable habitats. Future research could enhance the representation of ecological processes by incorporating individual behavioral observations, population dynamic models, and interaction network analyses. Furthermore, the current niche analysis was based mainly on macroclimatic variables, lacking integration with microhabitat characteristics (e.g., soil moisture, canopy structure) and microclimates [80]. This limits a deeper mechanistic understanding of species-habitat relationships. Subsequent research could combine remote sensing, field tracking experiments, population genomics, and landscape genetics to unravel the underlying mechanisms of gene flow and local adaptation behind the observed distribution patterns [81]. This integrated approach would improve the predictive robustness of models in real ecological contexts and provide a more comprehensive scientific basis for biodiversity conservation and biological control strategies.

## 5. Conclusions

This study used the biomod2 integrated model with optimized parameters to predict changes in the distribution of two important natural enemies—*Microstylum dux* and *M. oberthurii*—under current and future climate conditions. The results indicate that the suitable habitats of these two predatory robber flies are currently concentrated primarily in southeastern China. In the future, their distribution patterns will undergo a structural shift: high-suitability zones will expand, while moderate-suitability zones will shrink significantly, with the total area remaining relatively stable. The niche overlap between the two species will exhibit a complex dynamic pattern of “first divergence, then convergence, and finally divergence” in the future, suggesting that interspecific relationships will respond nonlinearly to climate change. These findings provide preliminary insights that may inform conservation considerations and the potential use of these robber flies as natural enemies, but caution is warranted because our correlative models do not account for prey availability, dispersal capacity, land use, or interspecific interactions. Future research should integrate multi-source observational data with population dynamics models to deepen understanding of species’ adaptation mechanisms to microhabitats. Additionally, incorporating non-climatic factors (e.g., vegetation, soil, human disturbance) would help develop more realistic regional allocation strategies for natural enemy resources, thereby supporting the sustainability of ecosystem services under climate change.

## Figures and Tables

**Table 1:** Environment variables retained after processing for *M. dux* and *M. oberthurii*.

Variable	Description	Unit	*M. dux*	*M. oberthurii*
bio2	Mean diurnal range	°C	√	√
bio8	Mean temperature of wettest quarter	°C	–	√
bio9	Mean temperature of driest quarter	°C	√	–
bio14	Precipitation of driest month	mm	√	–
bio15	Precipitation seasonality	–	–	√
bio19	Precipitation of coldest quarter	mm	√	√

Notes: √ indicates the environmental variable was retained for model construction of the corresponding species; – indicates the variable was excluded.

## Data Availability

The GBIF download DOIs for *Microstylum dux* and *Microstylum oberthurii* are GBIF Occurrence Download. Available online: https://doi.org/10.15468/dl.ugtj5c (accessed on 26 November 2025) and https://doi.org/10.15468/dl.smwms6 (accessed on 16 January 2026). The data that support the findings of this study are openly available in Mendeley Data at http://doi.org/10.17632/h98tdgpbg3.2 (accessed on 11 April 2026).

## Supplementary Materials

The following supporting information can be downloaded at https://www.mdpi.com/article/10.3390/insects17050533/s1, Figure S1: Heatmap of Pearson correlation coefficient (r) of 19 climatic factors; Figure S2: Evaluation results of (a–c) Maxent and (d–f) Maxnet models under different parameter combinations for *M. dux*; Figure S3: Evaluation results of Maxnet models under different parameter combinations for *M. oberthurii*; Figure S4: Relative importance of environmental variables in contributing to the habitat suitability for (a) *M. dux* and (b) *M. oberthurii* under current climatic conditions; Figure S5: Response curves of the key environmental variables influencing habitat suitability for (a,b) *M. dux* and (c,d) *M. oberthurii*; Figure S6: Comparison of niche breadth between *M. dux* and *M. oberthurii* under current and future climate scenarios under different periods and climate scenarios; Figure S7: Principal component analysis biplot of current climatic factors for *M. dux* and *M. oberthurii*; Figure S8: Principal component analysis score plot of *M. dux* and *M. oberthurii* under different periods and climate scenarios; Figure S9: Variable loading distributions of climatic factors under different periods and climate scenarios; Table S1: Performance evaluation of models for (a) *M. dux* and (b) *M. oberthurii* based on two evaluation metrics; Table S2: Statistical analysis of regional area Changes in habitat suitability ratings for (a) *M. dux* and (b) *M. oberthurii* under different periods and climate scenarios; Table S3: Summary of centroid migration distances and habitat ellipse metrics for (a) *M. dux* and (b) *M. oberthurii* under different periods and climate scenarios; Table S4: Mean multivariate similarity and proportion of negative values for (a) *M. dux* and (b) *M. oberthurii*; Table S5: Statistical Summary of the Ecological Niche Dynamics of *M. dux* and *M. oberthurii* under different periods and climate scenarios.

## Abbreviations

The following abbreviations are used in this manuscript:

SDMs	Species Distribution Models
GBIF	Global Biodiversity Information Facility
BCC-CSM2-MR	Beijing Climate Center climate system model version 2—medium resolution
SSP	Shared socioeconomic pathway
VIF	Variance inflation factor
ANN	Artificial Neural Network
CTA	Classification Tree Analysis
FDA	Flexible Discriminant Analysis
GAM	Generalized Additive Model
GBM	Generalized Boosting Model
GLM	Generalized Linear Model
MARS	Multiple Adaptive Regression Splines
MAXENT	Maximum Entropy
MAXNET	Maximum Entropy
RF	Random Forest
SRE	Surface Range Envelop
XGBOOST	eXtreme Gradient Boosting Training
AICc	Akaike information criterion correction
TSS	True Skill Statistic
ROC	Receiver Operating Characteristic
AUC	Area Under the Receiver Operating Characteristic Curve
L	Linear
Q	Quadratic
H	Hinge
P	Product
RM	Regularisation multiplier
FCs	Feature combinations
EMwmean	Weighted mean method
