# Supplementary material for: Predicting the Potential Geographic Distributions of Two Large Predatory Insects, Microstylum dux and M. oberthurii (Diptera: Asilidae), Under Climate Change: A Comprehensive Analysis Based on Optimised Biomod2 Ensemble Model
Source: Insects. 2026 May 21;17(5):533. doi: 10.3390/insects17050533 (PMC13207368; doi:10.3390/insects17050533)
Supplement: Supplementary file 1 [file insects-17-00533-s001.zip › insects-4288003-supplementary.pdf]

Supplementary materials

Table S1 Performance evaluation of models for (a) *M.dux* and (b) *M. oberthurii* based on two evaluation metrics.

Model	True Skill Statistic(TSS)	Area Under the Receiver Operating Characteristic Curve(AUC)
	(a)	
ANN	0.801	0.949
CTA	0.795	0.889
GAM	0.818	0.961
GBM	0.689	0.966
GLM	0.821	0.957
MARS	0.748	0.944
MaxEnt	0.569	0.786
Maxnet	0.836	0.964
RF	0.701	0.964
SRE	0.685	0.842
FDA	0.763	0.936
XGBOOST	0.781	0.952
Ensemble model	0.889	0.981
	(b)	
ANN	0.831	0.974
CTA	0.753	0.892
GAM	0.766	0.977
GBM	0.766	0.974
GLM	0.806	0.976
MARS	0.828	0.970
MAXENT	0.593	0.975
MAXNET	0.811	0.796
RF	0.726	0.975
SRE	0.584	0.792
FDA	0.771	0.959
XGBOOST	0.775	0.961
Ensemble model	0.887	0.988

Table S2 Statistical analysis of regional area Changes in habitat suitability ratings for (a) *M.dux* and (b) *M. oberthurii* under different periods and climate scenarios

Different Periods and Climate Scenarios	Total suitable area (×10^4km^2)	Percentage of area change (%)	Highly suitable area (×$10^4 km^2$)	Percentage of area change (%)	Moderately suitable area (×10^4km^2)	Percentage of area change (%)	Slightly suitable area (×10^4km^2)	Percentage of area change (%)
(a)								
Current	170.49	—	60.55	—	46.42	—	63.52	—
2050s_SSP1-2.6	181.85	6.66	72.63	19.96	38.67	-16.70	70.55	11.06
2070s_SSP1-2.6	170.95	0.27	82.60	36.43	28.98	-37.58	59.37	-6.54
2090s_SSP1-2.6	171.20	0.41	89.96	48.58	21.94	-52.73	59.30	-6.66
2050s_SSP2-4.5	172.32	1.07	77.44	27.90	34.18	-26.37	60.70	-4.44
2070s_SSP2-4.5	174.30	2.23	80.75	33.36	38.47	-17.14	55.08	-13.29
2090s_SSP2-4.5	165.52	-2.92	79.80	31.79	29.51	-36.43	56.21	-11.51
2050s_SSP5-8.5	177.12	3.89	82.37	36.04	32.09	-30.89	62.66	-1.35
2070s_SSP5-8.5	176.64	3.60	83.26	37.51	31.86	-31.38	61.53	-3.14
2090s_SSP5-8.5	176.92	3.77	73.91	22.06	35.81	-22.86	67.20	5.79
(b)								
Current	174.64	—	36.09	—	63.87	—	74.68	—
2050s_SSP1-2.6	168.18	-3.70	41.61	15.28	60.61	-5.10	65.96	-11.68
2070s_SSP1-2.6	173.43	-0.69	45.74	26.73	57.95	-9.26	69.74	-6.61
2090s_SSP1-2.6	175.54	0.51	39.84	10.38	56.17	-12.05	79.52	6.49
2050s_SSP2-4.5	161.82	-7.34	42.78	18.53	46.81	-26.71	72.24	-3.27
2070s_SSP2-4.5	170.94	-2.12	46.88	29.88	56.43	-11.65	67.64	-9.43
2090s_SSP2-4.5	181.20	3.76	40.69	12.74	60.65	-5.03	79.86	6.93
2050s_SSP5-8.5	178.79	2.37	42.06	16.52	56.39	-11.71	80.35	7.59
2070s_SSP5-8.5	168.96	-3.25	43.95	21.78	54.78	-14.23	70.23	-5.96
2090s_SSP5-8.5	169.81	-2.76	43.54	20.62	40.15	-37.14	86.13	15.33

Table S3 Summary of centroid migration distances and habitat ellipse metrics for (a) *M. dux* and (b) *M. oberthurii* under different periods and climate scenarios.

Different Periods and Climate Scenarios	Centroids				Ellipse		
	X Coordinate (°)	Y Coordinate (°)	Migration Distance (km)	Migration Direction (°)	Ellipse Area ($\times 10^4 km^2$)	Eccentricity	Major Axis Rotation (°)
				(a)			
Current	111.3707	28.8302	—	—	259.82	0.7382	178.88
2050s_SSP1-2.6	106.2999	30.6180	529.22	160.58	504.92	0.9026	178.68
2070s_SSP1-2.6	109.5971	28.6514	386.61	329.19	306.95	0.7929	180.78
2090s_SSP1-2.6	110.7285	28.9348	114.90	14.06	289.01	0.7598	180.49
2050s_SSP2-4.5	110.6980	28.7788	65.99	184.37	256.17	0.7605	183.83
2070s_SSP2-4.5	108.1569	29.7450	269.27	159.18	405.37	0.8434	178.76
2090s_SSP2-4.5	107.7307	28.7752	115.20	246.28	345.30	0.8531	181.17
2050s_SSP5-8.5	108.5053	29.4386	287.02	168.01	324.53	0.8332	182.12
2070s_SSP5-8.5	107.7611	28.9182	92.56	214.96	304.31	0.8527	180.29
2090s_SSP5-8.5	109.2399	28.8333	144.58	356.71	329.69	0.8061	181.75
				(b)			
Current	112.6086	28.0143	—	—	213.47	0.6926	186.61
2050s_SSP1-2.6	110.3394	28.0134	223.50	180.02	324.78	0.7651	183.78
2070s_SSP1-2.6	111.5306	27.7924	119.92	349.49	259.36	0.7247	186.68
2090s_SSP1-2.6	112.1372	27.5310	66.61	336.69	257.37	0.7032	192.50
2050s_SSP2-4.5	112.236	27.6342	55.98	225.60	231.78	0.6999	185.75
2070s_SSP2-4.5	111.7154	27.7735	53.75	165.02	216.69	0.7183	186.18
2090s_SSP2-4.5	111.2789	28.0785	54.74	145.06	264.39	0.7352	183.10
2050s_SSP5-8.5	111.5268	28.2307	109.17	168.68	280.61	0.7289	183.62
2070s_SSP5-8.5	111.6844	28.0031	29.64	304.69	215.67	0.7220	189.22
2090s_SSP5-8.5	111.2773	27.8691	42.79	198.23	350.73	0.7344	188.48

Table S4 Mean multivariate similarity and proportion of negative values for (a) *M. dux* and (b) *M. oberthurii*.

Different Periods and Climate Scenarios	Mean multivariate similarity	Percentage of cases with negative multivariate similarity
	(a)	
2050s_SSP1-2.6	30.3	0.02%
2070s_SSP1-2.6	29.5	0.02%
2090s_SSP1-2.6	29.9	0.02%
2050s_SSP2-4.5	31.4	0.02%
2070s_SSP2-4.5	29.5	0.02%
2090s_SSP2-4.5	34.7	0.05%
2050s_SSP5-8.5	31.7	0.03%
2070s_SSP5-8.5	29.6	0.03%
2090s_SSP5-8.5	33.0	0.09%
	(b)	
2050s_SSP1-2.6	18.9	0.24%
2070s_SSP1-2.6	18.6	0.28%
2090s_SSP1-2.6	18.2	0.09%
2050s_SSP2-4.5	18.6	0.18%
2070s_SSP2-4.5	18.6	0.28%
2090s_SSP2-4.5	16.3	0.21%
2050s_SSP5-8.5	16.8	0.21%
2070s_SSP5-8.5	18.6	0.28%
2090s_SSP5-8.5	11.2	3.24%

Table S5 Statistical Summary of the Ecological Niche Dynamics of *M. dux* and *M. oberthurii* under different periods and climate scenarios.

Different Periods and Climate Scenarios	*M. dux*		*M. oberthurii*		Ecological niche overlap	
	Niche breadth	Niche volume (95% core area)	Niche breadth	Niche volume (95% core area)	*D*	*I*
Current	0.257	0.346	0.539	0.747	0.464	0.764
2050s_SSP1-2.6	0.280	0.378	0.526	0.740	0.451	0.758
2070s_SSP1-2.6	0.290	0.402	0.534	0.740	0.518	0.806
2090s_SSP1-2.6	0.283	0.380	0.547	0.750	0.478	0.781
2050s_SSP2-4.5	0.275	0.366	0.547	0.755	0.457	0.761
2070s_SSP2-4.5	0.290	0.402	0.534	0.740	0.518	0.806
2090s_SSP2-4.5	0.313	0.431	0.573	0.786	0.496	0.797
2050s_SSP5-8.5	0.318	0.419	0.567	0.773	0.440	0.749
2070s_SSP5-8.5	0.290	0.402	0.534	0.740	0.518	0.806
2090s_SSP5-8.5	0.301	0.402	0.569	0.772	0.479	0.781

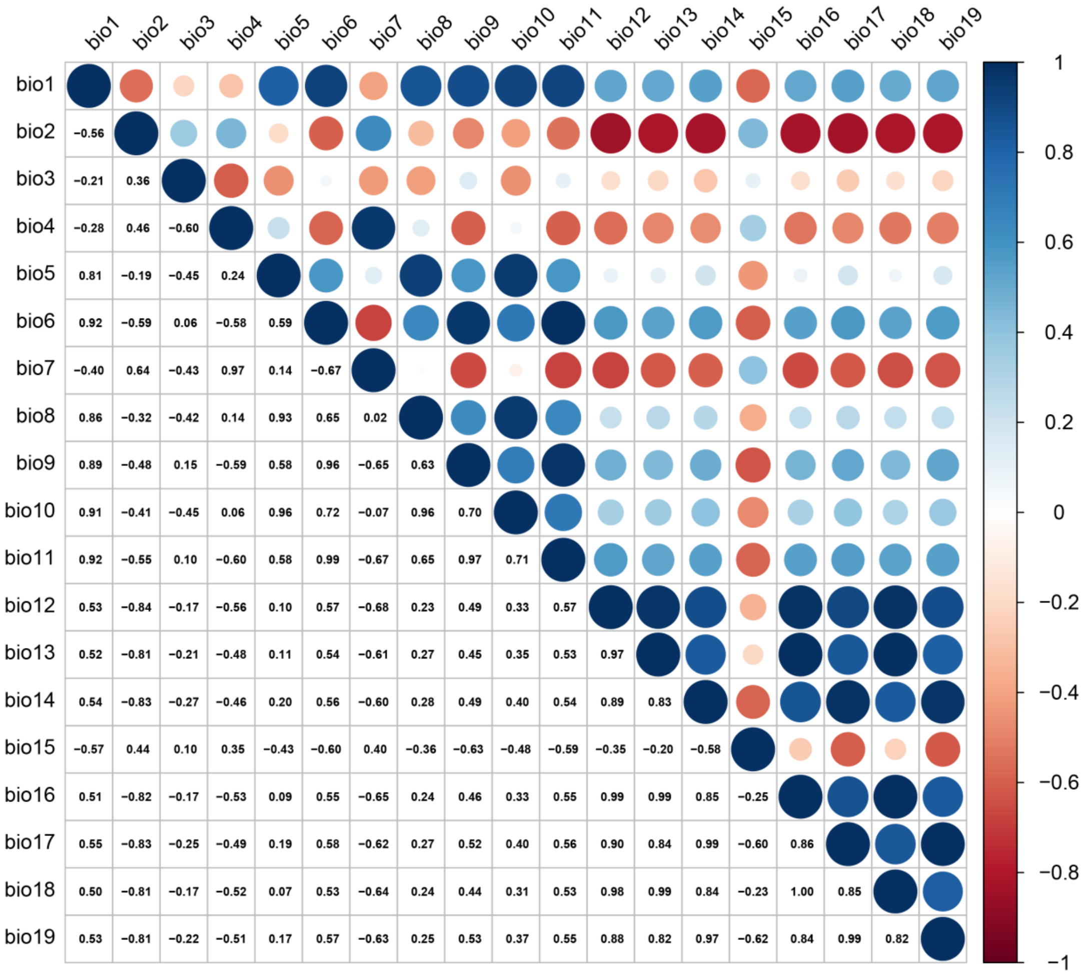

Figure S1 Heatmap of Pearson correlation coefficient (r) of 19 climatic factors

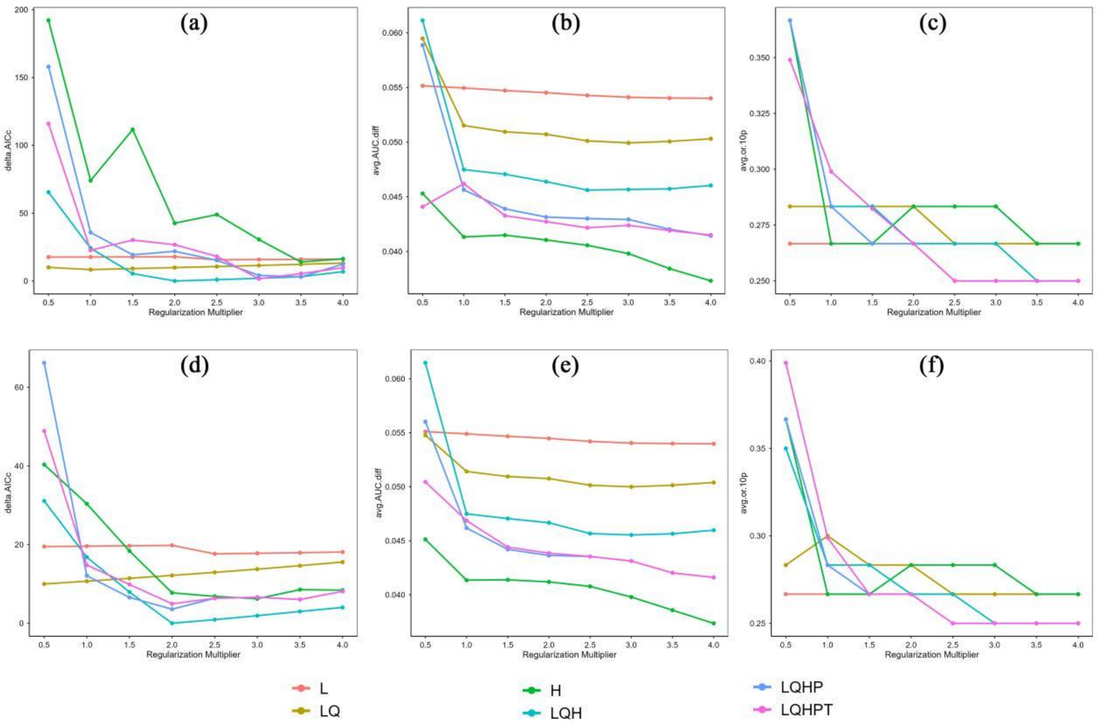

Figure S2 Evaluation results of (a-c) Maxent and (d-f) Maxnet models under different parameter combinations for *M.dux*

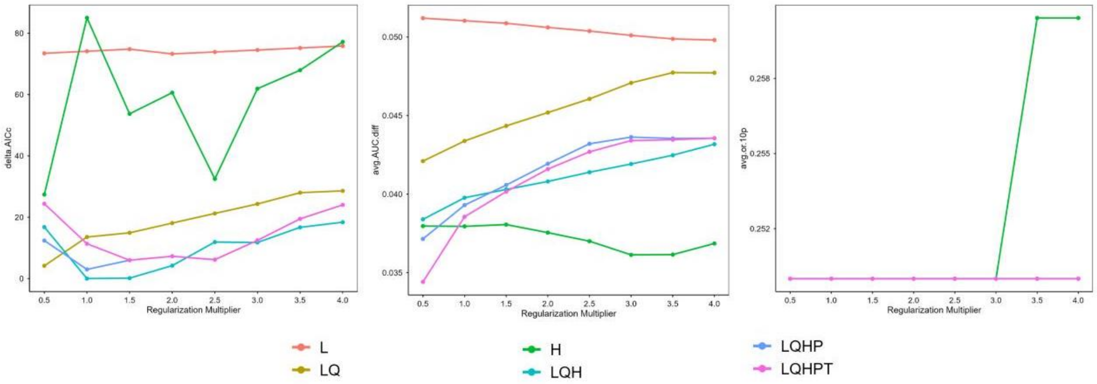

Figure S3 Evaluation results of Maxnet models under different parameter combinations for *M. oberthurii*

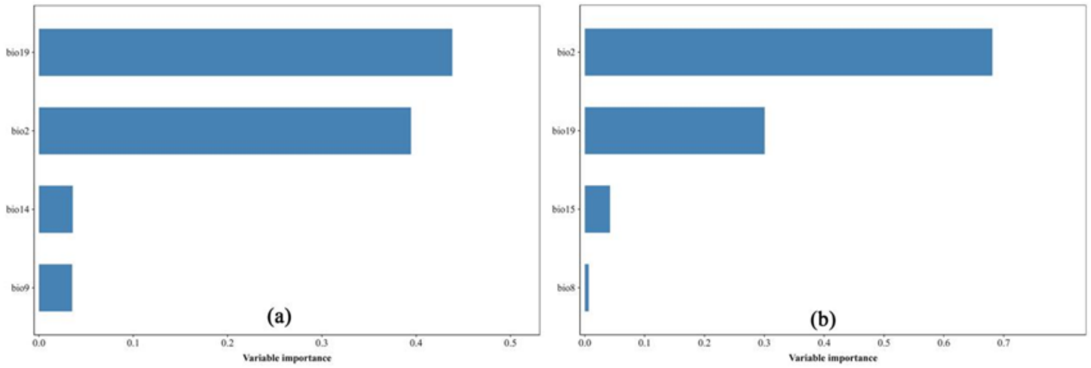

Figure S4 Relative importance of environmental variables in contributing to the habitat suitability for (a) *M. dux* and (b) *M. oberthurii* under current climatic conditions.

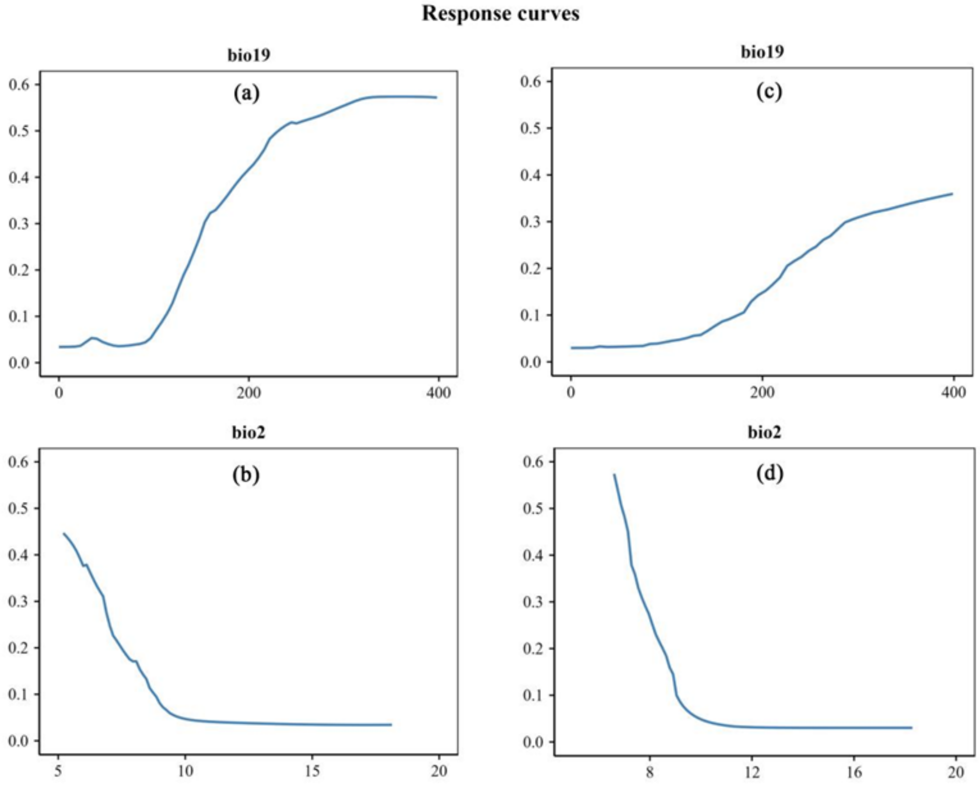

Figure S5 Response curves of the key environmental variables influencing habitat suitability for (a, b) *M. dux* and (c, d) *M. oberthurii.*

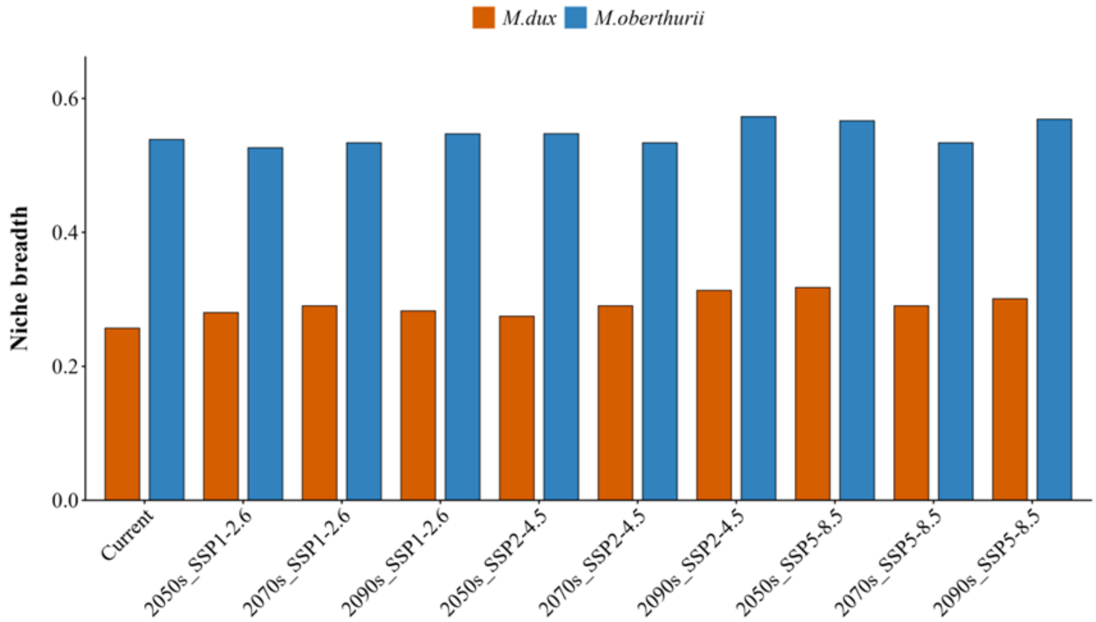

Figure S6 Comparison of niche breadth between *M. dux* and *M. oberthurii* under current and future climate scenarios under different periods and climate scenarios.

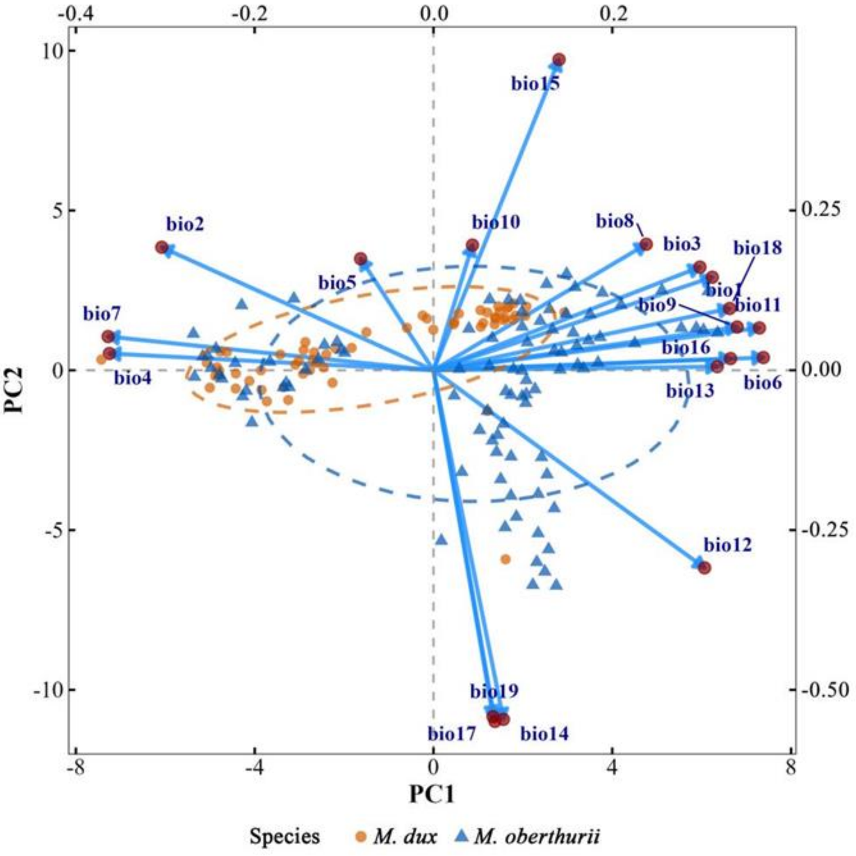

Figure S7 Principal component analysis biplot of current climatic factors for *M. dux* and *M. oberthurii.*

Note: The lower X-axis and left Y-axis represent species scores, while the upper X-axis and right Y-axis denote variable loadings.

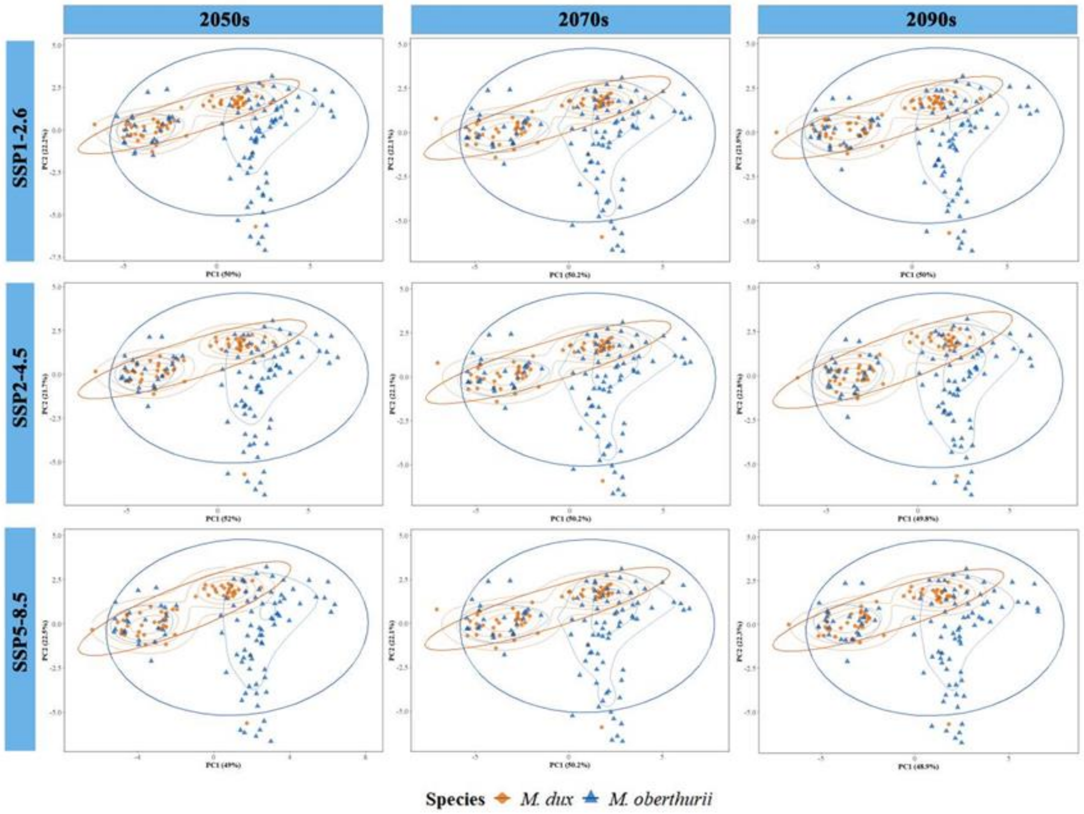

Figure S8 Principal component analysis score plot of *M. dux* and *M. oberthurii* under different periods and climate scenarios.

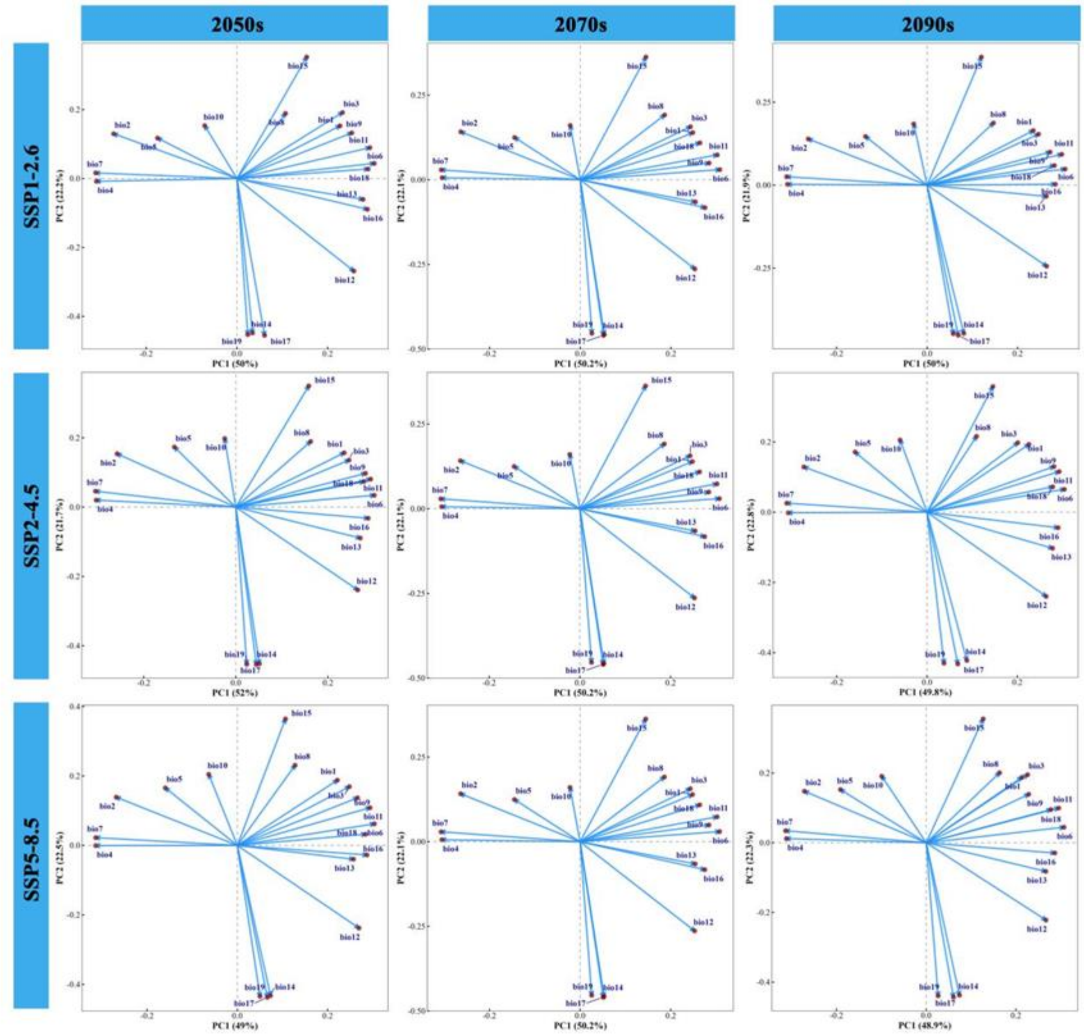

Figure S9 Variable loading distributions of climatic factors under different periods and climate scenarios.
